# Building for the future: essential infrastructure for rodent ageing studies

**DOI:** 10.1007/s00335-016-9646-7

**Published:** 2016-05-24

**Authors:** Sara E Wells, Ilaria Bellantuono

**Affiliations:** Mary Lyon Centre, MRC Harwell, Harwell Campus, Oxon, OX11 0RD UK; Department of Oncology and Metabolism, The Medical School, Beech Hill Road, Sheffield, S10 2RX UK

## Abstract

When planning ageing research using rodent models, the logistics of supply, long term housing and infrastructure provision are important factors to take into consideration. These issues need to be prioritised to ensure they meet the requirements of experiments which potentially will not be completed for several years. Although these issues are not unique to this discipline, the longevity of experiments and indeed the animals, requires a high level of consistency and sustainability to be maintained throughout lengthy periods of time. Moreover, the need to access aged stock or material for more immediate experiments poses many issues for the completion of pilot studies and/or short term intervention studies on older models. In this article, we highlight the increasing demand for ageing research, the resources and infrastructure involved, and the need for large-scale collaborative programmes to advance studies in both a timely and a cost-effective way.

## Introduction

Research using rodents as mammalian models for studying ageing are yielding considerable information on the genetic influences on longevity and, more recently, on the value of interventions targeting mechanisms of ageing to delay onset of age-related diseases (Check Hayden 2015; Kirkland 2013; Yuan et al. 2011). Until now, research on ageing and age-related diseases was considered separate fields of investigation with most research on ageing focusing on identifying influences on longevity, and research on age-related diseases centring on the study of mechanisms and treatment of a single disease. Often, mechanistic studies of diseases have been performed in young rodents and the effect of age on the disease pathogenesis or responses to interventions have not been considered. However, it is now clear that these approaches are far removed from the problems they are trying to solve. Age is the single largest risk factor for strokes, cardiovascular diseases, cancers, diabetes, and most other chronic diseases. In addition, approximately 60 % of those over 65 have been shown to have more than one condition at the same time, termed multimorbidity (Vogeli et al. 2007), and this is the main factor responsible for the decreased quality of life and increased health-care costs. Indeed, over 80 % of Medicare costs are related to age-related multimorbidity (Wolff et al. 2002). These patients often show decreased resilience and respond not so well to treatment. In addition, they show an increase in risk of serious side effects associated with polypharmacy (Marengoni et al. 2014; Tinetti et al. 2004). For these reasons, the *Population Level Commissioning for the Future* report from the UK government has recommended a more holistic approach to the care of age-related diseases, which moves away from the single disease approach (http://www.nhsiq.nhs.uk/news-events/news/population-level-commissioning-for-the-future.aspx).

With this in mind, scientists and geriatricians have proposed targeting research to the discovery of interventions which target common mechanisms of ageing to delay the onset of more than one age-related disease at the same time and improve health. This is based on evidence in preclinical murine models. Administration of interventions, such as dietary restriction (Fontana and Partridge 2015), rapamycin (Kaeberlein 2014), mefortmin (Pryor and Cabreiro 2015) or molecules which eliminate senescence cells (Chang et al. 2016; Zhu et al. 2015), has resulted in the delay of multiple age-related tissue dysfunction with improved cardiac function, cataracts, insulin sensitivity and reduced incidence of cancer. This suggests that delaying the onset of multimorbidity with a single agent may be achievable. However, the ability to conduct such preclinical intervention testing studies, which are scientifically robust and use suitable murine models and endpoints that are relevant to clinical translation not only poses technical challenges but also requires resources and interdisciplinary expertise.

In this article, we explore these challenges and propose that appropriate specialised infrastructures may be required to fast track those studies and reduce costs, both financial and in terms of animal usage.

### Models

The choice of animal models, which reproduce aspects of multimorbidity, frailty or loss of resilience and in which to accurately assess the effects of genes or molecules on healthspan, is still the subject of debate in the ageing research community. At present, the common practice is either to extrapolate data obtained from interventions in genetically homogeneous animal models or in disease models that develop the condition early in life. Yet, human populations are genetically heterogeneous and nearly all chronic diseases develop in humans over time and manifest later in life. In addition, no single cause is responsible for more than 25–30 % of human mortality. In contrast, in C57BL/6, the most common strain of mouse used for preclinical testing and to derive mutant mice at present, 86–89 % die of cancer (Blackwell et al. 1995). For comparison, cancer accounts for 23 % of the deaths in many developed countries (National Vital Statistics Report 2005).

In the USA, the intervention testing programme (ITP) at the NationaI Institute of Aging (NIA) has decided to use a four-way cross of different inbred mouse strains to increase genetic diversity. Whilst these mice have demonstrated a wider variety of conditions at death than C57BL/6, they are still far from the diverse disease phenotypes seen in humans (Nadon et al. 2008). Disease models often develop the condition early in life and spontaneously, in contrast to humans in whom nearly all chronic diseases develop over time and manifest in later life. Determining whether an intervention alters the onset of a disease in older mice is a complex task. This may require the introduction of a mutation which makes it susceptible to a disease. This is usually best achieved by generating conditional knock outs, which allow the manipulation of the onset of disease later in life. However, there are a limited number of such transgenic lines. The International Mouse Phenotyping Consortium (IMPC), aims to generate mouse lines and phenotypic analysis of knock out mouse mutants as models of disease (Brown and Moore 2012), but there has been little or no work to test the effect of age on disease development or the susceptibility of developing a second disease following the onset of the initial illness. Therefore, there is a real need to develop and properly characterise genetically modified animal models susceptible to multiple organ dysfunctions and chronic, later-onset diseases which can be challenged with stressors to test their resilience. This needs to be done using a standardised phenotypic pipeline of measurements, repeated over time in longitudinal studies. More importantly, it is unlikely that one model will recapitulate enough features of age-related multimorbidity or frailty to generate robust results to support clinical translation, and multiple models may be required. Testing in multiple models is resource intense and requires infrastructure with large capabilities.

### Reproducibility: strain, genetic integrity, environmental control and experimental design

There has been much publicity around the seemingly unreproducible data from animal studies. Undoubtedly the source of the lack of robustness in some rodent models is complex and multifactorial. However, there are emerging themes of strain, sex, genetic quality, environmental control and experimental design which are becoming even more pertinent when considering a study lasting over 2 years, with large investments in terms of both animal numbers and costs (Reardon 2016). Strain and sex are a source of great variability in outcomes in longevity studies. The introduction of 40 % dietary restriction to 41 different inbred strains of mice resulted in the predicted extension of lifespan in only 5 % of the strains in male and 21 % in female. Dietary restriction either had no effect or shortened lifespan in the other strains when compared to their respective ad libitum-fed littermates (Liao et al. 2010).

Extensive single nucleotide polymorphism (SNP) panels now enables more quality assurance of mouse strains than ever before as well as the opportunity to genetically screen stocks prior to their entry into ageing studies. Most mouse suppliers run genetic quality control programs to prevent gross contamination from other strains and limit the inevitable genetic drift. It is therefore relatively easy to access wild type mice of a high genetic integrity and importantly, of known genotype. The increasing wealth of phenotype data also makes it possible to exclude strains which carry mutations which may confound experimental data, such as C57BL/6 strains for hearing research as a mutation in cadherin 23 renders them deaf as they mature (Kane et al. 2012).

Controlling the genetic background of experimental cohorts with complex allele combinations is, however, more difficult. Intercrossing two strains of different genetic backgrounds will result in novel and different combinations of genes likely to modulate the effects of any genetic alterations to be studied. The challenge of ageing conditional transgenic models, with the prospect of lines bearing recombinase transgenes on different backgrounds than the conditionally engineered genes, poses issues not only in appropriate control cohorts but also in welfare and phenotypic issues of previously uncharacterised mixed genetic backgrounds. The generation of new mouse lines on genetically controlled coisogenic backgrounds using new genome editing techniques will inevitably reduce the variation caused by undefined background effects.

It is an obvious assumption that changes in environment over an extended period of housing has the potential to affect phenotypes and responses to intervention studies. A few decades ago it would have been predicted that this was limited to the composition of food and general environmental factors, such as temperature and humidity. However, extensive studies of how laboratory rodents are housed and the effect of differing conditions on phenotypes over the last few years have clarified a plethora of further considerations. Caging, environmental enrichment, feed, light intensity and housing density are all key factors which research facilities strive to keep constant (Tucci et al. 2006). It is the sustainability of a constant environment which is imperative to control ageing stock. This is not a trivial issue, and although changes such as altering the caging type within a facility happen infrequently which are usually accompanied by an extensive planning exercise, smaller changes such as the use of alternate environmental enrichment or food supplements needs to be fastidiously controlled. To facilitate constant and uncompromised conditions for ageing research, it is ideal to have dedicated colonies/rooms or facilities which are specifically designed for sustained, constant, environmental conditions and where the smallest of alterations in the housing of ageing stock is discussed widely, recorded comprehensively and the impact of which is assessed extensively. The difficulties of accounting for all of these variables is exemplified by the experience of the National Institute of Health (NIH) which has recognised the importance of reaching consensus on these practises and has established a centralised intervention testing programme (NIA ITP). Despite standardisation of some of these variables, testing of aspirin, nitroflurbiprofen (NFP), 4-OH-alpha-phenyl-*N*-tert-butyl nitrone (4-OH-PBN), or nordihydroguaiaretic acid (NDGA) resulted in large variability in survival (Strong et al. 2008). Among the possible causes was the type of food used for the breeding stock or for weaning the mice prior to the start of the intervention.

High mortality rates from infections, such as mouse norovirus (Kastenmayer et al. 2008) or intermittent infections of diseases which are likely to alter immune status, can greatly confound the analysis of the effect of genetic alterations or interventions on mouse healthspan. The aspiration for most modern animal facilities is for specific pathogen free (SPF) status achieved by regular negative test results for a list of pathogens recommended by organisations such as FELASA (Mahler Convenor et al. 2014). The microbiological status of animals entering, during and indeed completing ageing research programs are important metadata to be collected through rigorous screening and used in subsequent analysis.

### Measuring healthspan

Although lifespan is a more commonly used endpoint, healthspan is much more desirable from a clinical perspective. However, healthspan is more difficult to assess and requires well equipped infrastructures with multidisciplinary expertise. A number of healthspan measures have emerged to assess the state of multiple organs, physiological systems and behaviour in mice. Measures of physical performance, cognitive function, body composition, immune function, sensory acuity, and metabolic state have been proposed as they are clinically relevant and capture important aspects of ageing. The International Mouse Phenotyping Consortium has published a database of standard operating procedures that can be used to phenotype a mouse (www.mousephenotype.org). This has been used mainly to phenotype young mice but it is an excellent starting point for discussion on which tests best measure healthspan across systems and ages and reflect clinical outcomes. Investigators from a number of US institutions came together to discuss what is the best way of assessing health and their recommendation can be found in Richardson et al. (2016) where strength and limitations of current pipelines for the measurement of healthspan are highlighted. Resilience in older age, defined as the ability to better cope with adversity, is also considered a measure of health. A number of clinically relevant perturbations and insults to model stressors have been proposed (i.e. chemotherapy, anaesthesia, pneumonia, hip fracture, surgery, sepsis, hypovolemia, cold exposure and wound healing), together with the rate of recovery and tolerance to the perturbation assessed by measures such as food intake, body weight, grooming, habitual physical activity or other physiological variables. However, these tests present considerable welfare issues, particularly in older mice and therefore at present few centres have the capability to introduce them and they remain largely untested. In addition there is a need to agree whether all these measurements are better performed in longitudinal study to comprehensively assess the natural history of changes in measures of healthspan. Understanding the onset, order, rate and magnitude of changes in parameters of healthspan may foster better design, execution and interpretation of studies of geroprotectors.

### Going forward

The cost and capability to perform long-term genetic, mechanistic and intervention studies using statistically significant number of animals followed for long periods of time (24–30 months) assessed by multiple tests is high. According to power calculations executed by the NIA ITP, each intervention should be tested in 44 male and 36 female mice, and this should be repeated in at least three independent sites (Nadon et al. 2008). This type of approach focuses only on lifespan as an endpoint. If healthspan needs to be used, this will require monitoring of a very high number of parameters, the responses to challenges in more than one strain or disease model or in modified conditions to mimic exposure to factors such as tobacco or alcohol, excess nutrients. This will increase the number of animals, technology and infrastructure required. Many research laboratories do not have the resources, equipment or the expertise to conduct scientifically robust intervention studies of this kind on their own. Moreover, scientists tend to have a single discipline approach whereas these type of approaches require multidisciplinary expertise. Considering the costs for the necessary instrumentation to perform many of the measures required, the personnel and organisational infrastructure and wide-ranging expertise required, the possibility of creating centralised testing facilities, would make economic sense. Centralisation would help avoid duplication and ensure testing is rigorous and comparable across laboratories, according to a well-established pipeline, which would promote fast translation to the clinic. This would also allow better planning for the acquisition of older animals for the testing programme and donation of unused tissues for mechanistic studies or more specialised assessment outside the pipeline. Stocks of young wild rodents are easily purchased from commercial breeders while more complex genetically modified strains are accessible from international repositories through the International Mouse Strain Resource (IMSR: www.findmice.org). In both cases the starting material of live animals or germplasm are readily available for experiments. However, for studies requiring aged tissue, sometimes into the geriatric range of over 2 years, there are limited supplies from commercial breeders. This is understandable for both ethical and cost reasons. It would not be morally acceptable to maintain animals in laboratories ‘just in case’ they were required. Furthermore, matching supply and demand in fast moving academic fields is difficult and each mouse of this age typically costs in the region of several hundreds of pounds to house to over 2 years. Solutions to these issues must lie in large collaborative exercises, where aged stocks can be utilised in the most comprehensive way possible, serving a number of research projects whilst sharing ageing resources. While in the USA the NIH has been promoting this for some time with the NIA ITP and the Aged Rodent Tissue bank, such infrastructures are in their infancy in Europe. An example is the Shared Ageing Research Models (www.Sharmuk.org), which has developed a tissue bank for the collection of unused tissues from investigators willing to donate the tissues and a database of live ageing colonies of mice made available from investigators who use only one or two tissues and are willing to donate the remaining tissues to interested investigators for their bespoke collection. This is encouraging but more is required if we are to meet the societal challenge of an increasing ageing population.
